# Clinical characteristics and outcomes of intracranial hemorrhage in geriatric patients receiving non–vitamin K antagonist oral anticoagulants: a multicenter observational study

**DOI:** 10.1007/s11845-026-04404-0

**Published:** 2026-04-21

**Authors:** Ramazan Guven, Akkan Avci, Ozgur Sogut, Serkan Dogan, Mehmet Akif Karamercan, Ertugrul Altinbilek, Semih Korkut, Ertugrul Altug, Begum Seyda Avci, Yunus Dogan, Adem Az, Gokhan Eyupoglu, Said Kural, Ogulcan Alp, Metin Ozmen, Ozgur Guven, Yeliz Simsek, Erdem Aksay, Ahmet Burak Urfalioglu, Pelin Oguz Tura, Hakan Kaan Yilmaz, Sara Tunca, Can Ilcin, Efe Demir Bala, Munisahan Güvercin

**Affiliations:** 1 Department of Emergency Medicine, Faculty of Medicine, Atlas University Hospital, Istanbul, Turkey; 2 Department of Emergency Medicine, Adana City Research and Training Hospital, Health Science University, Adana, Turkey; 3 Department of Emergency Medicine, Haseki Research and Training Hospital, University of Health Sciences, Istanbul, Turkey; 4 Department of Emergency Medicine, Kanuni Sultan Suleyman Research and Training Hospital, University of Health Sciences, Istanbul, Turkey; 5 Department of Emergency Medicine, Gazi University Faculty of Medicine, Gazi University Hospital, Ankara, Turkey; 6 Department of Emergency Medicine, Hamidiye Sisli Etfal Research and Training, University of Health Sciences, Istanbul, Turkey; 7 Department of Emergency Medicine, Basaksehir Cam and Sakura City Hospital, University of Health Sciences, Istanbul, Turkey; 8 Department of Internal Medicine, Adana City Research and Training Hospital, University of Health Sciences, Adana, Turkey

**Keywords:** NOACs, Intracranial hemorrage, Mortality, Geriatric patients

## Abstract

**Background:**

Evidence comparing different non–vitamin K antagonist oral anticoagulants (NOACs) in terms of intracranial hemorrhage (ICH) characteristics and clinical outcomes is limited.

**Aim:**

This study aimed to identify factors associated with mortality and morbidity in geriatric patients receiving different NOACs presenting to the emergency department with intracranial hemorrhage.

**Methods:**

This multicenter retrospective observational study. Geriatric patients (aged ≥ 65 years) who presented to the emergency department with ICH while receiving NOAC therapy were included. Clinical characteristics were extracted from electronic medical records. The primary outcome was in-hospital mortality, while secondary outcomes included hemorrhage volume, intraventricular hemorrhage, intensive care unit admission, and the effect of four-factor prothrombin complex concentrate administration. Statistical analyses were performed using the chi-square or Fisher’s exact tests for categorical variables and the Mann–Whitney U or t-test for continuous variables, with *p* < 0.05 considered statistically significant.

**Results:**

A total of 47 patients were included; 57.4% (*n* = 27) were female and 42.6% (*n* = 20) were male. No significant association was observed between NOAC type and in-hospital mortality (*p* = 0.533). Intraventricular hemorrhage was significantly more frequent in the rivaroxaban group than in the apixaban group (42.9% vs. 9.5%, *p* = 0.014). Intraventricular hemorrhage (52.6% vs. 4.2%, *p* < 0.001) and atrial fibrillation (90.5% vs. 57.7%, *p* = 0.012) were significantly more common in the mortality group compared with the survival group.

**Conclusion:**

NOAC type was not associated with in-hospital mortality; however, intraventricular hemorrhage occurred more frequently in patients receiving rivaroxaban and was significantly associated with mortality.

## Introduction

Non–vitamin K antagonist oral anticoagulants (NOACs) are a group of drugs used to prevent stroke and systemic emboli in patients with atrial fibrillation (AF), coronary artery disease (CAD), or venous thromboembolism (VTE) [1]. The use of NOACs has steadily increased since their debut, for they have a wide therapeutic index, no interaction with food, and high patient compliance than Vitamin K antagonists (VKAs). In 2015, NOACs accounted for one-third of oral anticoagulant drugs [2]. In some countries, use of NOACs has increased as high as 80% versus VKAs as of 2022. However, their relatively high cost, risk of bleeding, and lack of sufficient clinical evidence about their specific antidotes raise concerns about their use [3]. The most feared complication of anticoagulation is intracerebral hemorrhage (ICH), as it is responsible for most of the morbidity and mortality resulting from anticoagulant-associated bleeding. ICH has been found to occur in 0.1–0.2% of patients receiving NOACs each year [4]. In addition, a 27% high in-hospital mortality rate has been reported in patients with NOAC-associated spontaneous intracerebral hemorrhage [5]. Advanced age, large hematoma volumes, and hematoma width are accused reasons for the high mortality rate [4]. This is due to both increased burden of comorbidity, such as hypertension, diabetes mellitus, or cardiovascular diseases, and age-related changes in brain structures among geriatric patients [6].

However, studies directly comparing the effects of different NOACs on ICH and comparing the differences in clinical outcomes of these drugs are scarce. This multicenter study evaluated characteristics of ICH, treatment approaches, and clinical outcomes in geriatric patients receiving NOACs. The aim of the study was to determine factors affecting mortality and morbidity in geriatric patients receiving different types of NOACs presenting with ICH to the emergency department.

## Methods

### Study design and setting

This multicenter study, designed as a retrospective observational study, includes 5 hospitals that are among the largest hospitals in their region. The coordinating center was located in Istanbul, and the other hospitals were in Ankara and Adana. Necessary permissions were obtained from all centers, and the study was approved by the local ethics committees with number KAEK/26.06.2024.53 (Basaksehir Cam and Sakura City Hospital Clinical Research Ethics Committee). Since this was a retrospective study, informed patient consent was not obtained. Our study was conducted according to the retrospectoscope guidance of Kaji et al. (2014), which aims to reduce bias for chart review studies in the emergency department [7].

### Patient selection

Geriatric patients (age ≥ 65 years) who were admitted to the emergency department with ICH under NOAC treatment between the years 2022 and 2024 were included in the study. The exclusion criteria for the study were incomplete medical or imaging records and trauma. Statistical data for the 47 patients included in the study were analyzed.

### Data sources

Patients diagnosed with ICH were identified using ICD-10 (International Classification of Diseases 10th Revision) codes. During screening, ICD-10 codes were used for subarachnoid hemorrhages (I60.0-I60.9, including carotid siphon, middle cerebral artery, anterior and posterior communicating arteries, basilar and vertebral arteries), intracerebral hemorrhages (I61-I61.9, subcortical, cortical, brainstem, cerebellar, intraventricular, and multiple localizations), and other non-traumatic intracranial hemorrhages (I62.0, I62.1, I62.9, acute subdural, extradural, and unspecified intracranial hemorrhages). Only patients diagnosed with these ICD-10 codes, meeting the criteria, and aged 65 years and older were included in the study.

### Data collection

Data were extracted from electronic medical records, including demographics (age, gender), comorbidities (hypertension, diabetes mellitus, atrial fibrillation, cardiovascular disease), vital signs (systolic/diastolic blood pressure, heart rate), and laboratory values (prothrombin time (PT), and activated partial thromboplastin time (aPTT), and International Normalized Ratio (INR)). Radiological data from computed tomography (CT) scans documented hemorrhage type, location (subdural, epidural, subarachnoid, intraventricular), and volume measured using the ABC/2 method, as described by Kothari et al. [8]. ABC/2 scoring was performed by two independent radiologists who were blinded to the study hypothesis and patient data. The measurement results were determined by the consensus of both researchers and then provided to the data analysis team.

### Outcomes and definitions

The primary outcome was in-hospital mortality. Secondary outcomes included hemorrhage volume at admission, intraventricular hemorrhage, ICU admission, and the impact of four-factor prothrombin complex concentrate (4 F-PCC) administration in the ED on hemorrhage progression and outcomes.

Patients in the standard care group followed routine protocols like IV fluids, blood pressure management, and symptomatic treatments such as antiemetics or anticonvulsants, but they did not receive 4 F-PCC.

### Statistical analysis

The data were analyzed with SPSS version 28.0 (IBM Corp.) statistical software. Continuous variables were reported as means ± SD (standard deviation) or medians with interquartile ranges (IQR), and categorical variables as counts and percentages. Chi-square or Fisher’s exact tests were used for categorical data and Mann-Whitney U or t-tests for continuous variables. The Monte Carlo exact test was applied for small samples. A p-value < 0.05 was considered statistically significant.

## Results

A total of 47 patients were included in the study; 57.4% (*n* = 27) were female and 42.6% (*n* = 20) were male. The mean age of the included patients was 79.1 ± 6.4 years. Clinical characteristics of the patients according to the anticoagulant use are shown in Table 1. An equal number of patients were using rivaroxaban (*n* = 21) and apixaban (*n* = 21), whereas two patients were using dabigatran and three patients were using edoxaban.

**Table 1:** Clinical characteristics of patients on direct oral anticoagulants

	Rivaroxaban (*n* = 21) 15 mg (*n *= 4) 20 mg (*n* = 17)	Apixaban (*n* = 21) 2.5 mg (*n* = 12) 5 mg (*n* = 9)	Edoxaban (*n* = 3)	Dabigatran (*n* = 2)
Gender, n(%)				
*Female*	13 (61.9)	12 (57.1)	1 (33.3)	1 (50.0)
*Male*	8 (38.1)	9 (42.9)	2 (66.7)	1 (50.0)
Age, years (± s.d.)	76.3 (6.9)	82.1 (4.5)	74.6 (8.0)	83.0 (1.4)
AF, n(%)	14 (66.7)	16 (76.2)	1 (50.0)	1 (50.0)
HT, n(%)	17 (81.0)	16 (76.2)	3 (100)	2 (100)
DM, n(%)	6 (28.6)	9 (42.9)	2 (66.7)	1 (50.0)
CVD, n(%)	12 (57.1)	12 (57.1)	3 (100)	1 (50.0)
SBP, mmHg (± s.d.)	150.3 ± 29.1	146.5 ± 40.8	149.6 ± 21.4	146.0 ± 48.0
DBP, mmHg (± s.d.)	84.4 ± 21.1	83.3 ± 19.6	92.0 ± 8.1	104.0 ± 8.4
HR, bpm (± s.d.)	80.9 ± 19.3	81.6 ± 13.6	83.0 ± 15.7	97.5 ± 3.5
PT, seconds median (IQR)	13.6 (9.1)	14.5 (27.3)	20.3	96.5
aPTT, seconds median (IQR)	31.7 (11.6)	30.1 (11.1)	27.7	44.0
INR level, median (IQR)	1.4 (0.8)	1.1 (0.8)	1.8	7.5
Using FFP	9.5 (2)	1 (50.0)	0 (0.0)	4.8 (1)
Using 4F-PCC in ED, n(%)	3 (14.3)	8 (38.1)	0 (0.0)	1 (50)
Outcome, n(%)				
*Hospital admission*	6 (28.6)	9 (42.9)	0 (0.0)	1 (50)
	*15 mg: 1*	*2.5 mg: 5*		
	*20 mg: 5*	*5 mg: 4*	1 (50)	0 (0.0)
*ICU admission*	5 (23.8)	4 (19.0)	2 (66.7)	1 (50)
	*15 mg: 1*	*2.5 mg: 3*		
	*20 mg: 4*	*5 mg: 1*		
*In-hospital Mortality*	10 (47.6)	8 (38.1)		
	*15 mg: 2*	*2.5 mg: 4*		
	*20 mg: 8*	*5 mg: 4*		

*AF,* Atrial Fibrillation; *HT,* Hypertension; *DM,* Diabetes mellitus; *CVD,* cardiovascular disease; *SBP,* Systolic Blood Pressure; *DBP,* Diastolic Blood Pressure; *HR,* Heart Rate; *PT,* Prothrombin Time; *aPTT,* activated partial thromboplastin time; *INR,* International Normalized Ratio; *FFP,* Fresh Frozen Plasma; *4F-PCC,* Four-factor prothrombin complex concenrate; *ED,* Emergency Department; *ICU,* Intensive Care Unit

When the volume and localization of ICH, occurrence of intraventricular hemorrhage, and in-hospital mortality were compared between two groups on the most commonly used NOACs, apixaban and rivaroxaban, only the occurrence of intraventricular hemorrhage was observed to be significantly higher in the rivaroxaban group than the apixaban group (9.5% vs. 42.9%; *p* = 0.014). There was no significant difference between both agents in other parameters evaluated (Table 2).

**Table 2:** Comparison of hemorrhage localization, volume and in-hospital mortality between Apixaban and Rivaroxaban with significance values

	Apixaban (*n* = 21)	Rivaroxaban (*n* = 21)	*p*-value
*Localization of hemorrhage,			
Subdural hematoma	4 (19.0)	6 (28.6)	
Epidural hematoma	2 (9.5)	0 (0.0)	0.170
Diffuse subarachnoid hemorrhage	1 (4.8)	5 (23.8	
Other locations	14 (66.7)	10 (47.6)	
Intraventricular hemorrhage, n (%)	2 (9.5)	9 (42.9)	0.014
Volume of intracerebral hemorrhage in ED, ml median (IQR)	19.3 (23.0)	21.7 (28.9)	0.770
In-hospital mortality, n (%)	8 (38.1)	10 (47.6)	0.533

*ED,* Emergency Department

*Monte Carlo Exact Test was used

The baseline intracerebral hemorrhage volumes at admission to the emergency department (ED) based on cranial CT scans, the percentage reduction of intracerebral hemorrhage volumes between the initial CT scan in the ED and the CT scan in the ward, and the in-hospital mortality by standard care approach and 4 F-PCC arms were compared (Table 3). According to the analysis, the percentage reduction of intracerebral hemorrhage volumes between the first CT scan in the ED and the CT scan in the ward was found to be significantly higher in the 4 F-PCC group than in the standard care group (76.5% vs. 36.0%, *p* = 0.028). In-hospital mortality rate by standard care group was more than double the in-hospital mortality rate by 4 F-PCC arm, but this difference was not statistically significant (51.4% vs. 25.0%, *p* = 0.112).

**Table 3:** Comparison of hemorrhage volume, volumetric change and in-hospital mortality between 4F-PCC and standard care group with significance values

	Administration of 4F-PCC (*n* = 12)	Standard Care Group (*n* = 35)	*p*-value
Volume of intracerebral hemorrhage at admission, ml median (IQR)	10.9 (54.7)	23.0 (28.6)	0.530
*Volumetric change of intracerebral hemorrhage, % median (IQR)	76.5 (34.0)	36.0 (79.0)	0.028
In-hospital mortality, n(%)	3 (25.0)	18 (51.4)	0.112

*4F-PCC,* Four-factor prothrombin complex concentrate; *IQR,* interquartile range

* Percentage reduction of intracerebral hemorrhage volumes between the first CT scan in the ED and the first CT scan after hospitalization. There was no standardization regarding the timing of CT in the ED and after hospital admission

Clinical characteristics of the patients included in the study were examined in two groups as survival and mortality (Table 4). AF was significantly higher in the mortality group than in the survival group (90.5% vs. 57.7%, *p* = 0.012). The presence of intraventricular hemorrhage was found to be significantly more frequent in the mortality group than in the survival group (52.6% vs. 4.2%, *p* < 0.001).

## Discussion

The most important finding of this study was that the rate of intraventricular hemorrhage was significantly higher in patients receiving rivaroxaban compared to patients receiving apixaban. In addition, the application of 4 F-PCC in the emergency department was found to provide a significant reduction in hematoma volume compared to standard care group. Although the in-hospital mortality rate by standard care group was more than double the in-hospital mortality rate by 4 F-PCC, this difference was not statistically significant.

Specific antidotes for NOACs were not available in our country as of 2025. 4 F-PCC is recommended for hemorrhage related with NOACs if specific antidotes are not available [9]. 4 F-PCC is also selected for patients with small-sized intracerebral hemorrhage in this study. The drivers of treatment selection to manage ICH should be further investigated in a separate multicenter study with questionnaires.

**Table 4:** Comparison of clinical characteristics and hemorrhagic findings between survival and mortality

	Survival (*n* = 26)	Mortality (*n* = 21)	*p*-value
Gender, n (%)			
Female	14 (53.8)	13 (61.9)	0.579
Male	12 (46.2)	8 (38.1)	
Age, years (± Sd)	78.3 (±6.9)	80.2 (±5.8)	0.284
SBP, mmHg (± Sd)	140.2 (±26.6)	158.2 (±39.6)	0.060
DBP, mmHg (± Sd)	81.5 (±15.0)	89.8 (±23.6)	0.091
HR, bpm (± Sd)	80.5 (±15.1)	83.9 (±17.8)	0.472
HT, n (%)	21 (80.8)	17 (81.0)	0.987
DM, n (%)	10 (38.5)	8 (38.1)	0.980
AF, n (%)	15 (57.7)	19 (90.5)	0.012
CVD, n (%)	17 (65.4)	11 (52.4)	0.366
Volume of intracerebral hemorrhage in admission, ml median (IQR)	9.8 (40.4)	23.1 (12.6)	0.082
Intraventricular hemorrhage, n (%)	1 (4.2)	10 (52.6)	<0.001

*AF,* Atrial Fibrillation; *bpm,* beat per minute; *CVD,* cardiovascular disease; *DBP,* Diastolic Blood Pressure; *DM,* Diabetes mellitus; *HR,* Heart Rate; *HT,* Hypertension; *IQR,* interquartile range; *SBP,* Systolic Blood Pressure; *Sd,* Standard Deviation

Our study was conducted in a population consisting entirely of patients aged 65 years and older. It is important because the rate of anticoagulant use is higher and bleeding complications are more severe in the geriatric patient group. Geriatric population is also prone to increased rate of falls or accidents that are a risk factor for ICH [10]. Changes in cranial structures, fragility, accompanying comorbidities, polypharmacy, and metabolic changes that may potentially impact the pharmacokinetics of used drugs in geriatric patients could be some of the factors that increase the risk of ICH in this population. Therefore, it is important to examine the results of bleeding related to NOAC use in this patient group in more detail. Our findings showed that the rate of intraventricular hemorrhage in this patient group was significantly higher in patients using rivaroxaban and that this may increase the risk of mortality. Medication errors on elderly population were commonly reported [11], and it could be a problem on NOAC users [12]. In patients with AF, it is estimated that someone would need to fall 45 times a year for rivaroxaban or 458 times a year for apixaban for the risk to outweigh the benefit [13]. Considering the risk‑to‑benefit ratio, apixaban should be the preferred NOAC rather than rivaroxaban.

NOACs are increasingly preferred to prevent thromboembolic events, especially in patients with AF. However, management of ICH, which is the most serious complication of these agents, is a major problem in clinical practice. There is evidence in the literature that apixaban is safer than rivaroxaban [14]. In 2023, the expert panel of American Geriatrics Society updated Beers Criteria^®^ for potentially inappropriate medication use in older adults, “The recommendation for rivaroxaban has changed from ‘use with caution,’ to ‘avoid’ for long-term treatment of nonvalvular AF and VTE, with the rationale being that observational studies and network meta-analyses find that this drug confers a higher risk of major and gastrointestinal (GI) bleeding in older adults than other DOACs, particularly apixaban but also dabigatran. Comparison of rivaroxaban with apixaban in geriatric population is a hot topic and investigators are encouraged to publish their data [15]. We think that our study makes an important contribution as one of the limited number of studies evaluating the differences between these drugs. Our findings showed that the rate of intraventricular hemorrhage was higher in patients using rivaroxaban, suggesting that this drug should be used more carefully in certain patient groups like geriatric patients 65 years or older.

Previous studies have shown that apixaban reduces intracerebral hemorrhage by 31% and hemorrhage-related mortality by 11% [16]. Although it has been suggested that rivaroxaban provides similar benefits, some studies have reported that rivaroxaban causes more complications [17]. This situation was confirmed in our study and it was shown that intraventricular hemorrhage was significantly higher in the rivaroxaban group. This difference may be due to the short half-life of rivaroxaban and fluctuations in plasma concentrations that may occur due to its use as a single daily dose. It is thought that rivaroxaban, which cannot reach sufficient levels in plasma, increases the risk of intraventricular spread of hemorrhage because it cannot provide effective clotting control. In a phase I study in subjects with moderate renal impairment (CrCl 30–49 mL/min), the use of rivaroxaban was associated with an increase in plasma concentration (1.5-fold increase in the area under the plasma concentration-time curve [AUC]). Additionally, age > 75 years (1.4-fold increase in AUC) and moderate hepatic impairment (2.3-fold increase in AUC) may lead to higher exposure. The risk of bleeding is potentially increased when rivaroxaban is used with other anticoagulants [18]. An increased risk of bleeding should be considered when rivaroxaban is administered with other anticoagulants.

4F-PCC, used in the management of bleeding, has the potential to improve bleeding control by rapidly reversing the anticoagulant effect. Previous studies have shown that 4 F-PCC application reduces hematoma expansion and provides hemostatic efficacy [19, 20] In our study, a significant reduction of the hematoma volume was observed in patients who received 4 F-PCC in the emergency department compared to patients who received standard care treatment. However, no statistically significant difference was found between the two groups in terms of in-hospital mortality rates. This may be explained by the relatively small patient size and the inability to control other factors that may affect mortality (general health status of the patients, primary location of the bleeding, etc.). However, it suggests that early application of 4 F-PCC may be clinically important, especially in terms of limiting hematoma expansion.

Our study showed that one of the most important predictors of mortality in geriatric patients with NOAC-associated ICH was the presence of intraventricular hemorrhage. As is known, intraventricular hemorrhage is an important determinant for the risk of mortality, independent of the volume and location of hemorrhage. AF was also found to be significantly associated with mortality, which is consistent with the available data in the literature. Since AF patients are generally older and might have several comorbidities. For this reason, these patients are expected to have a worse prognosis after ICH.

### Limitations

Although our study has a multicenter design, and data from different hospitals increased generalizability by covering a wider range of the population, it had some limitations. First, the retrospective design contained the risk of selection bias that may affect the accuracy of the data. The relatively small patient size limited the statistical power, especially in the evaluation of secondary outcomes such as mortality rates. In addition, the lack of standardization of variables such as the timing of 4 F-PCC administration and dose differences of 4 F-PCC may make it difficult to interpret the results. Differences in the timing of CT scans also limited the objective assessment of hemorrhage volume changes. Lastly, due to the very small number of patients using Edoxaban and Dabigatran in our cohort, a detailed comparison of outcomes across all NOAC types could not be performed.

## Conclusion

This study showed that the occurrence of intraventricular hemorrhage was significantly higher in geriatric patients with suspected NOAC-related ICH in those receiving rivaroxaban. Administration of 4 F-PCC was found to significantly reduce the volume of hemorrhage but had no significant effect on in-hospital mortality. These findings emphasize the importance of early intervention with 4 F-PCC in the management of NOAC-related hemorrhage and suggest that clinicians should be more careful in the selection of NOACs. Future larger-scale, prospective studies should be conducted to further evaluate the effects of different NOACs on the hemorrhage profile in the geriatric patient group.
